# A new strategy for TiO_2_ whiskers mediated multi-mode cancer treatment

**DOI:** 10.1186/s11671-015-0796-4

**Published:** 2015-02-28

**Authors:** Peipei Xu, Ruju Wang, Jian Ouyang, Bing Chen

**Affiliations:** Department of Hematology, The Affiliated Drum Tower Hospital of Nanjing University Medical School, Nanjing, 210008 People’s Republic of China; Medical School, Southeast University, Nanjing, 210009 People’s Republic of China

**Keywords:** Titanium dioxide whiskers, Gambogic acid, Photodynamic therapy, Drug delivery system, Leukemia, 61.46. + w, 8783, 8760 F

## Abstract

Traditional Chinese medicine (TCM) which functions as chemotherapeutic or adjuvantly chemotherapeutic agents has been drawing a great many eyeballs for its easy obtain and significant antitumor effects accompanied with less toxic and side effects. PDT (photodynamic therapy) utilizes the fact that certain compounds coined as photosensitizers, when exposed to light of a specific wavelength, are capable of generating cytotoxic reactive oxygen species (ROS) such as hydroxyl radical, hydrogen peroxide, and superoxide to kill cancer cells. Combinations of cancer therapeutic modalities are studied to improve the efficacy of treatment. This study aimed to explore a new strategy of coupling of titanium dioxide whiskers (TiO_2_ Ws) with the anticancer drug gambogic acid (GA) in photodynamic therapy. The nanocomposites were coined as GA-TiO_2_. The combination of TiO_2_ Ws with GA induced a remarkable enhancement in antitumor activity estimated by MTT assay, nuclear DAPI staining, and flow cytometry. Furthermore, the possible signaling pathway was explored by reverse transcription polymerase chain reaction (RT-PCR) and Western blot assay. These results identify TiO_2_ Ws of good biocompatibility and photocatalytic activity. In human leukemia cells (K562 cells), TiO_2_ Ws could obviously increase the intracellular concentration of GA and enhance its potential antitumor efficiency, suggesting that TiO_2_ Ws could act as an efficient drug delivery carrier targeting GA to carcinoma cells. Moreover, photodynamic GA-TiO_2_ nanocomposites could induce an evident reinforcement in antitumor activity with UV illumination. These results reveal that such modality combinations put forward a promising proposal in cancer therapy.

**Electronic supplementary material:**

The online version of this article (doi:10.1186/s11671-015-0796-4) contains supplementary material, which is available to authorized users.

## Background

Chemotherapy plays a fundamental role in the treatment for the most majority of cancers, including leukemia. It has been well documented that traditional Chinese medicine (TCM), as a crucial constituent member of complementary and alternative medicine, has been widely used in the preventive and therapeutic treatment for a variety of cancers during the last decades [1-3]. Gambogic acid (GA, C_38_H_44_O_8_), isolated from gamboge resin obtained from the *Garcinia hanburyi* tree [4-6], is one of such recently discovered TCM. Whereas evident antitumor properties has been addressed to date in a plethora of carcinoma cell lines, the present clinical application of GA is plagued by its serious side effects, such as phlebophlogosis, myelosuppression, and oral ulcers [7].

To maximize antitumor effects and lower undesirable side effects of GA associated with high dosages of chemotherapeutic agents, new therapies in cancer treatment applying nanoparticle-based drug delivery systems are being developed and have recently drawn substantial experimental interest.

Titanium dioxide whiskers (TiO_2_ Ws), which are commonly used in many biomedical studies including promoting tissue repair, drug delivery, cell separation, tissue targeting, transfection, and cellular/molecular tracking, have been well elucidated till now that they are not just proved to be environmental friendly but also possess exclusive photocatalytic activity, which can generate reactive oxygen species (ROS) upon UV irradiation [8-12]. These properties tend to allow TiO_2_ Ws to be used in photodynamic therapy (PDT), which highly potentiates the tumor targeting of chemotherapeutic agents and elicits ROS formation, leading to cellular destruction eventually [13].

Based on these observations, we developed new GA-TiO_2_ nanocomposites to facilitate the properties of GA, in which TiO_2_ Ws were initially coated with GA. In this study, we estimated the cytotoxicity and efficiency in PDT, examined the effectiveness of antitumor activity in K562 cells, and investigated the antitumor mechanism of the nanocomposites, suggesting that TiO_2_ Ws could not only be applied as the drug delivery system to carry GA into cancer cells but could also improve the antitumor effects for synergistic PDT.

## Methods

### Materials

GA (molecular formula, C38H44O8; Kanion Pharmaceutical Co. Ltd, Jiangsu, People’s Republic of China) was dissolved in dimethyl sulfoxide (DMSO; Sigma-Aldrich, St Louis, MO, USA), stored at −20°C, and then suspended as needed in Roswell Park Memorial Institute medium (RPMI) (1640, Life Technologies, Carlsbad, CA, USA). The cytotoxicity assay kit was obtained from were obtained from Sigma-Aldrich (St Louis, MO, USA) and stored in the dark. RT-PCR kit was from Takara Biotechnology (Dalian, China). Horseradish peroxidase-conjugated IgG antibodies, caspase-3, CDK2, CDK4, cyclin D1, p21 and p27, and β-actin monoclonal antibodies were purchased from Nanjing KeyGen Biotech Co., Ltd. (Nanjing, People’s Republic of China). All the other regents used in this report were analytical pure.

### Preparation and characterization of TiO_2_ Ws

TiO_2_ Ws were prepared as described previously [14,15]. TiO_2_ Ws were finally obtained with the tetragonal crystal structure (anatase). To characterize the microstructure of TiO_2_ Ws nanomaterial, the transmission electron microscope (TEM) images were obtained using a JEM-2100 transmission electron microscope (JEOL, Tokyo, Japan) at room temperature (20°C ± 2°C). The sample morphology was evaluated by scanning electron microscopy (SEM, JEOL JSM-5900). The crystalline phase was determined by powder X-ray diffraction (XRD) pattern (Bruker D8, Cu-Ka radiation) obtained on a DMAX-B (Rigaku Denki Corp, Tokyo, Japan). N_2_ adsorption-desorption measurements were employed to study the textural properties at liquid nitrogen temperature (TriStar II 3020, Norcross, GA, USA).

### Cytotoxicity evaluation

Every experiment was repeated at least three times. Ultrasonic treatments of TiO_2_ Ws for about 30 to 50 min were performed in the following experiments. K562 and HELF cells in log phase were trypsinized and seeded in 96-well plates. After 24 h of incubation, the cultured cells were rinsed in Dulbecco’s Modified Eagle’s medium and incubated with different concentrations of TiO_2_ Ws (1.56, 3.13, 6.25, 12.5, and 25 ug · mL^−1^) for 6 h at 37°C in the dark. Upon application of UV irradiation (*λ* = 254 nm), in which the average intensity was 0.1 mW · cm^−2^ at the working plane, the effect of TiO_2_ Ws for K562 cell proliferation in the presence of UV irradiation for 180 s was investigated. After treatment, the cells were returned to the incubator for 24 h. The MTT solutions were added into it, and the mixtures were incubated for another 4 h. DMSO was added to solubilize the formazan crystals, and OD 570 was recorded.

### Preparation of GA-TiO_2_ nanocomposites and the characterization of GA loading and release

GA solution was diluted by PBS (pH 7.4, 0.01 M) or mixed into TiO_2_ Ws suspension (10 ug mL^−1^) with various concentration (0.125, 0.25, 0.5, 1, 2, 4 ug mL^−1^). They were kept respectively below 4°C for more than 24 h in the dark to enable the GA to conjugate with TiO_2_ Ws.

To allow the loading and estimation of the drug encapsulation efficiency, GA was separated from GA-TiO_2_ nanocomposites through centrifugation at 15,000 rpm for 30 min, and the supernatant was determined by HPLC. Then, the loading efficiency (LE) and encapsulation efficiency (EE) were calculated as the following equations:

$$ \mathrm{loading}\ \mathrm{efficiency} = \left(\mathrm{amount}\ \mathrm{of}\ \mathrm{drug}\ \mathrm{in}\ \mathrm{drug}\hbox{-} \mathrm{loaded}\ \mathrm{nanocomposites}\ /\ \mathrm{amount}\ \mathrm{of}\ \mathrm{drug}\hbox{-} \mathrm{loaded}\ \mathrm{nanocomposites}\right)\ 100\% $$

$$ \mathrm{encapsulation}\ \mathrm{efficiency} = \left(\mathrm{amount}\ \mathrm{of}\ \mathrm{drug}\ \mathrm{in}\ \mathrm{drug}\hbox{-} \mathrm{loaded}\ \mathrm{nanocomposites}\ /\mathrm{initial}\ \mathrm{amount}\ \mathrm{of}\ \mathrm{drug}\right)\ 100\% $$

The releasing capacity of GA from GA-TiO_2_ nanocomposites was investigated at pH 6.0 (pH of the environment around the tumor), and pH 7.4 (pH of physiological blood). In brief, the GA-TiO_2_ nanocomposites were dispersed in PBS (pH 7.4, 5 mL) and transferred into the dialysis bag. The dialysis bag was immersed in 95 mL PBS of pH 6.0 and 7.4, respectively. Then, the release medium was continuously agitated with stirring speed 100 rpm at 37°C. Two milliliters of the external medium was collected and replaced with the same fresh PBS at predetermined time intervals. The amount of released GA in the medium was analyzed by HPLC.

### Cell culture

K562, human chronic myelogenous leukemia cells, were obtained from the Institute of Hematology at the Chinese Academy of Medical Sciences (Beijing, People’s Republic of China). HELF, human embryonic lung fibroblast cells, were obtained from the Shanghai Institute of Cells at the Chinese Academy of Sciences (Shanghai, People’s Republic of China). They were maintained in RPMI 1640 medium supplemented with 10% heat-inactivated newborn bovine serum (Sigma-Aldrich), 100 U/mL penicillin, and 100 mg/mL streptomycin at 37°C in a humidified atmosphere with 5% CO_2_ and passaged once every 2 to 3 days.

### *In vitro* cytotoxicity assays

K562 cells were seeded at 2 × 10^4^ cells/well in a 96-well plate and administered with different concentrations of GA in solution (GA-Sol) or GA-TiO_2_ after 6 h. The doses of GA incorporated in TiO_2_ share the same concentration with GA-Sol. The culture medium was replaced with 200 mL of three groups of medium containing free GA (0, 0.125, 0.25, 0.5, 1, 2, and 4 μg/mL), GA-TiO_2_ nanocomposites, or GA-TiO_2_ nanocomposites with 180 s of UV irradiation, at 6 h of cell culture. After irradiation, the cell lines were returned to the incubator for 24 h. The relative cytotoxicities of the three groups were assessed by MTT assay. Microscope was employed for investigating the morphological of cells.

### DAPI staining

The cells were treated as the above three groups of methods for 24 h and then were fixed with 4% polyoxymethylene prior to washing with PBS. The washed cells were then stained with 1 mg/mL DAPI for 15 min in the dark. The staining images were seen and observed under the fluorescent microscope.

### Flow cytometric apoptosis assay

FACSCalibur flow cytometry (Becton Dickinson, Franklin Lakes, NJ, USA) was employed to test the apoptosis of K562 cells which were treated in different systems. In short, 4 × 10^5^ K562 cells were washed after exposing to GA, TiO_2_ Ws, GA-TiO_2_ composites, or GA-TiO_2_ composites (UV) for 24 h. Subsequently, 500 uL of binding buffer was added and mixed with 5 udL of Annexin V-FITC; the mixture was kept at room temperature for 15 min in the dark. Flow cytometry analyses were performed using CellQuest software to determine the apoptosis of cells, in which the excitation wavelength was 488 nm and the emission wavelength was 530 nm, over 1 h.

### Reverse transcription polymerase chain reaction (RT-PCR) assay

The RT-PCR method was employed to determine the transcription levels of genes at the transcription level. The experimental procedures were carried out according to the conventional methods. The designed PCR primers were shown in Table 1.

**Table 1:** **The designed PCR primers of genes**

**Gene**	**Primer**
Caspase-3	sense 5′-GTGCTATTGTGAGGCGGTTGT-3′
	antisense 5′-TGAGGTTTGCTGCATCGACAT-3′
CDK2	sense 5′-CATTCCTCTTCCCCTCATCA-3′
	antisense 5′- GTCACCATTTCGGCAAAGAT −3′
Survivin	sense 5′-TGTAAGTGCCATCTGGTAGC-3′
	antisense 5′-ATGCGCCAGTTTCTAAGAGG-3′
Cyclin D1	sense 5′-CCGTCCATGCGGAAGATC-3′
	antisense 5′-CCTCCTCCTCGCACTTCTGT-3′
P21	sense 5′-CCCGTGGACAGTGAGCATGG-3′
	antisense 5′-ATGGAGGAGCCGGGACGA-3′
P27	sense 5′-CAGAATCATAAGCCCCTGGA-3′
	antisense 5′-TCTGTTCTGTTGGCCCTTTT-3′
GAPDH	sense 5′-TGTTGCCATCAATGACCCCTT-3′
	antisense 5′-CTCCACGACGTACTCAGCG-3′

### Western blot assay

The K562 cells were treated with the above methods for 24 h and then centrifuged at 10,000 rpm for 5 min. Western blotting was done as a usual way. After normalization with the corresponding expression of β-actin, the expression levels of apoptosis regulatory proteins (e.g., caspase-3, CDK2, CDK4, cyclin D1, p21, and p27) were determined using densitometry scans.

### Statistical analysis

All data were presented as mean ± standard deviation (SD) of three identical experiments. Statistical significance of the differences was determined using Student’s *t*-test by means of SPSS software (version 13.0; SPSS Inc, Chicago, IL, USA). *P* values <0.05 were considered as statistically significant.

## Results and discussion

### Characterization of TiO_2_ Ws

**Figure 1:**
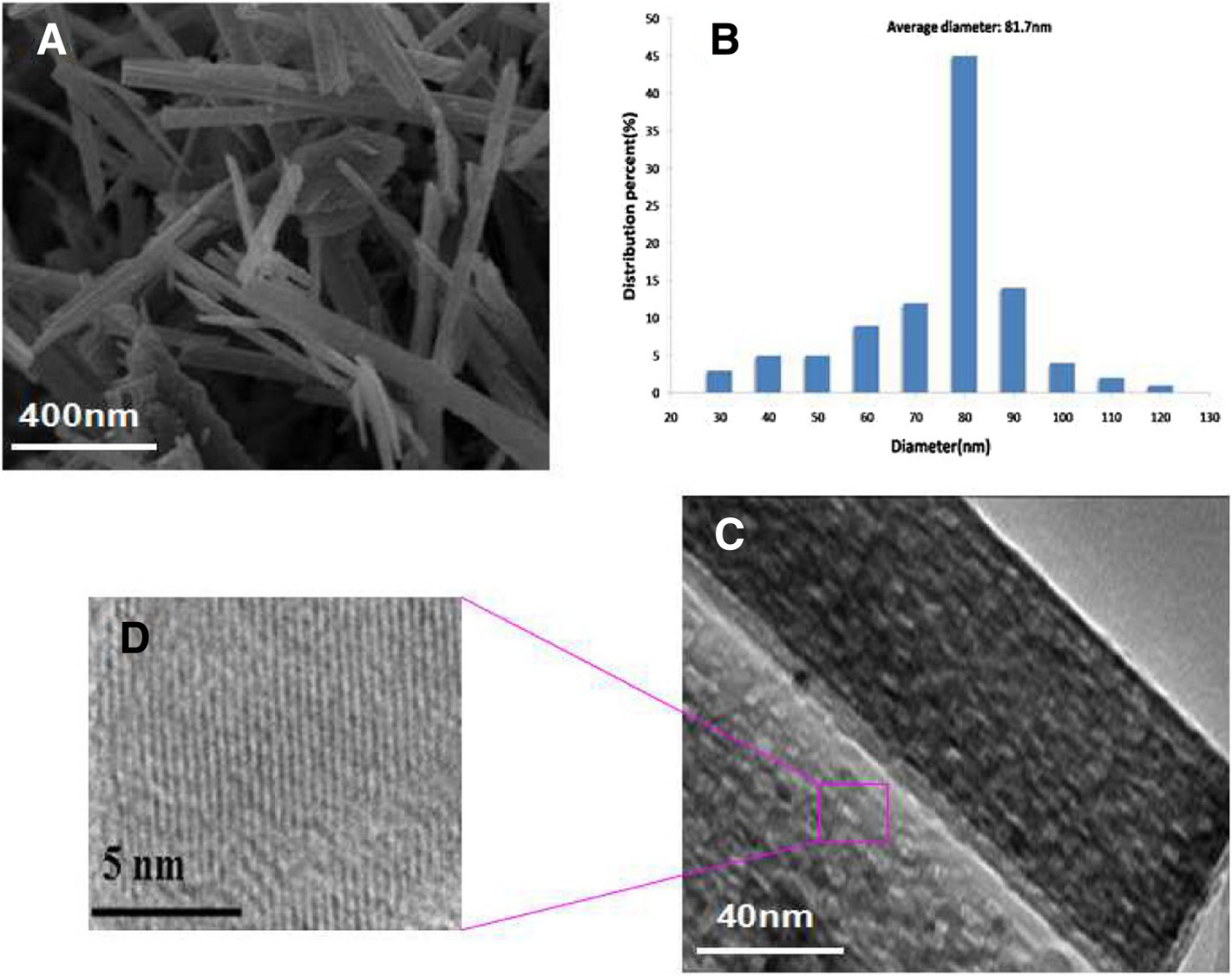
**Characterization of TiO**_**2**_ **Ws.** Notes: **(A)** SEM image (left) and **(B)** diameter distribution (right). **(C)** Typical TEM micrograph of a region (left), and **(D)** high magnification of image (right).

**Figure 2:**
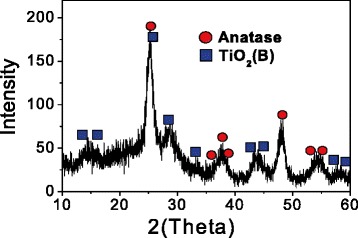
**XRD patterns of TiO**_**2**_ **Ws (gray lines: anatase phase JCPDS).**

The typical TEM images of the GA-TiO_2_ nanocomposites are shown in Figure 1. The TiO_2_ nanostructures exhibited a needle-like morphology, with an average size of 81.7 nm approximately in width and 200 to 1,000 nm in length. These data indicate that TiO_2_ Ws have the uniform diameter distribution. The crystalline nature of the as-prepared TiO_2_ was analyzed based on its X-ray diffraction (XRD) pattern as displayed in Figure 2. The diffraction peaks were quite consistent with those of bulk TiO_2_, which could be indexed as TiO_2_ (JCPDF 35–0088). Sharp peaks were observed, suggesting that the nanostructures possessed large crystalline domains and a high degree of crystallinity. No other peaks related to impurities were detected in the XRD patterns, confirming the purity of the synthesized TiO_2_ Ws. Nanomaterials could be divided into four kinds of types including nanoparticles, nanofibers, nanofilm, and nanobulk. TiO_2_ nanofibers had better properties of photocatalysis, suggestive of biomedical application in cancer therapy [16]. TiO_2_ Ws which we prepared is a kind of chopped nanofiber with a high degree of monocrystalline. These findings identify the former one as a better photocatalytic agent.

### Cytotoxicity testing and the application of TiO_2_ whiskers in PDT

**Figure 3:**
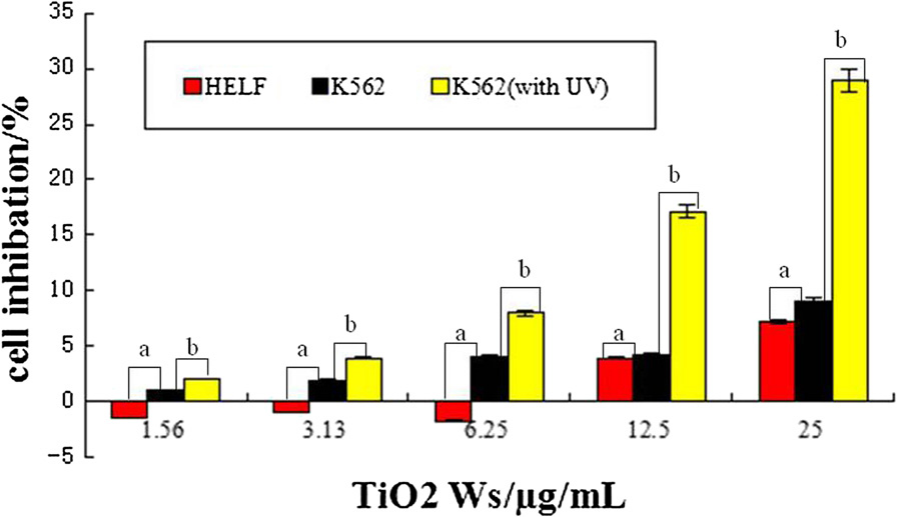
**The cytotoxicity of TiO**_**2**_ **Ws for K562 and HELF cells at 24 h** ***in vitro*** **.** Notes: **(a)** The cytotoxicity of TiO_2_ Ws for K562 and HELF cells (P > 0.05); **(b)** The cytotoxicity of TiO_2_ Ws (no UV) and TiO_2_ Ws (with UV) for K562 cells (P < 0.05).

Cytotoxicity tests tend to be carried out prior to biomedical application. The cytotoxicity of TiO_2_ Ws on K562 and HELF (human embryonic lung fibroblast) cells was measured by MTT assay. About 95% of the cells survived after treatment with TiO_2_ Ws at concentrations of up to 12.5 μg/mL, indicating low cytotoxicity (Figure 3). The lack of cytotoxicity of TiO_2_ Ws suggests the potential of applications in the fields of biomedical science and cancer therapy. Accordingly, we chose 10.0 ug/mL TiO_2_ Ws for subsequent studies.

Then, the photocatalytic activity of TiO_2_ Ws was estimated on cancer cells. K562 cells which were treated with the combination of TiO_2_ Ws and UV irradiation elicited a remarkable enhancement of mortality compared with cells only treated with TiO_2_ Ws, indicating the photocatalytic activity of TiO_2_ Ws (Figure 3). We then observed that treatment with TiO_2_ Ws resulted in an increase of lethality on cancer cells in a dose-dependent manner.

PDT has emerged as an alternative and promising noninvasive treatment for cancer [17]. PDT utilizes the fact that certain compounds coined as photosensitizers, when exposed to light of a specific wavelength, are capable of generating cytotoxic ROS such as hydroxyl radical, hydrogen peroxide, and superoxide to kill cancer cells [18,19]. TiO_2_, ZnO, and other semiconductor nanomaterials are regarded as the potential photosensitizing agents among the photosensitizers for PDT, due to their unique phototoxic effect upon the irradiation. They have been used in the treatment of cutaneous squamous cell and basal cell carcinomas as well as cancers of the head, neck, lung, esophagus, and bladder [20]. Compared to other photosensitizers, TiO_2_ Ws which we prepared are bio-safe and non-cytotoxic, leading to priority application in clinical chemotherapy. TiO_2_ Ws, therefore, can be one of the promising nanomaterials.

### Loading efficiency and *in vitro* drug release behavior

**Figure 4:**
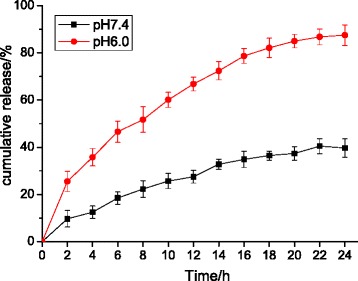
***In vitro*** **GA release behaviors at pH 7.4 and 6.0 respectively.**

The EE and LE of GA-TiO_2_ nanocomposites were assessed and calculated as 74.53% ± 5.43% and 15.11% ± 2.36%, respectively, showing a promising option of TiO_2_ Ws-loaded GA to act as an anticancer drug delivery carrier. It could be seen from Figure 4 that the release of drug molecules was dependent on the pH of the medium, as well as the releasing time. Within 24 h, the drug release ratio was 36% at pH 7.4, which was slow and sustained, whereas at pH 6.0, the GA release rate was much faster, with approximately 87.5%. As mentioned above, the clinical application of GA has been limited because of its serious side effects. The result suggests that the side effects to the normal tissues could be greatly reduced due to the prolonged GA retention time in blood circulation, which was down to the pH-triggered release behavior, namely in the environment of pH 7.4. In the normal physiological conditions, most GA is hypothesized to remain in the carrier for a considerable time. Once the GA-TiO_2_ nanocomposites are taken up by cancer cells via endocytotic process, a faster release may occur at lower local pH, i.e., surrounding the tumor site or inside the endosome and lysosome of tumor cells, causing a tremendous development in cancer treatment nowadays.

### Antitumor activity *in vitro*

Accumulating data show that GA has a broad spectrum of antitumor activity against diverse tumor cells, such as human multiple myeloma U266 cells, human lung carcinoma SPC-A1 cells, and human hepatoma SMMC-7721 cells [21,22]. This potential anticancer activity *in vitro* and *in vivo* is mainly attributed to the downregulation of telomerase activity and induction of the apoptotic process [23,24]. However, serious side effects of GA impede its clinical application. The combined application of TiO_2_ and PDT reduce the concentration of GA, thus lowering down the side effect.

**Figure 5:**
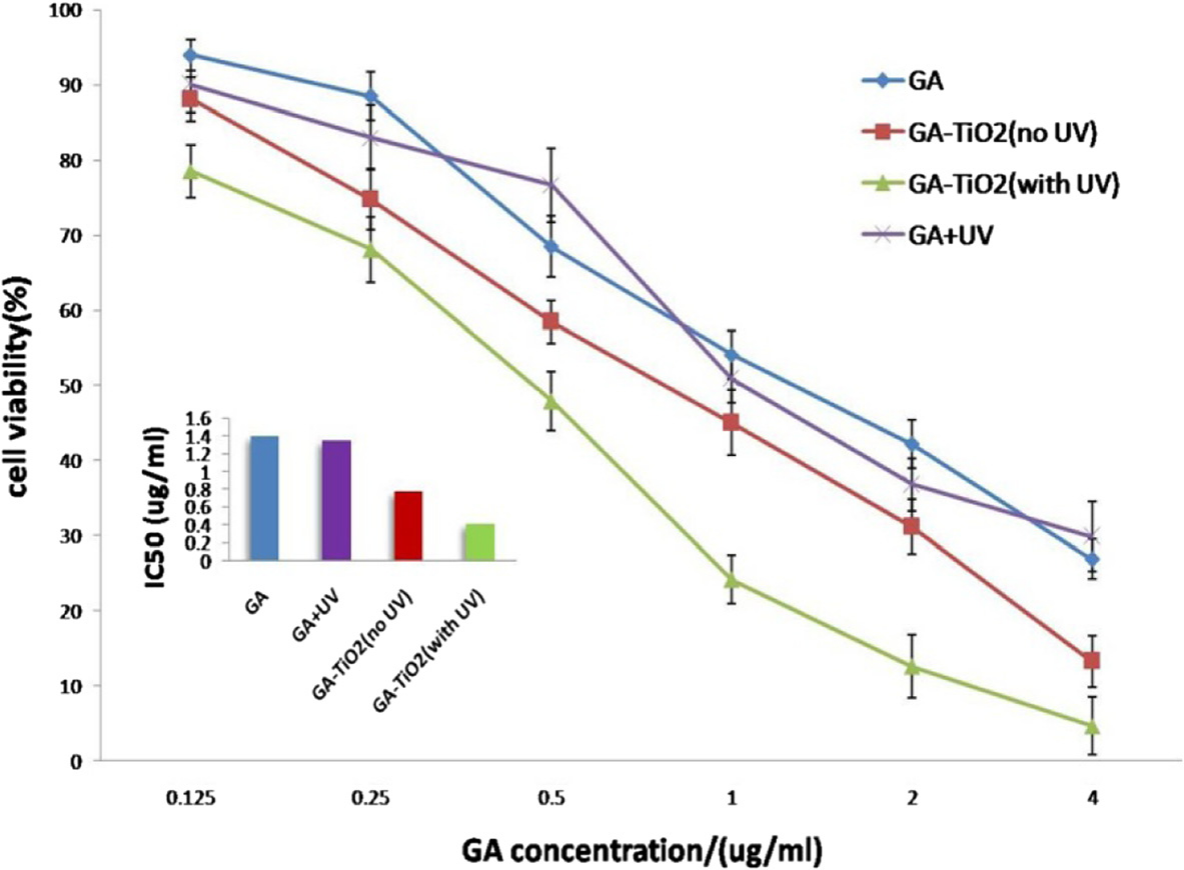
**Cytotoxic effect of GA or GA-TiO**_**2**_ **nanocomposites with/no UV.** Cytotoxic effect of GA or GA-TiO_2_ nanocomposites in the absence or presence of UV irradiation against K562 cells. Inset: the IC_50_ of GA and GA-TiO_2_ nanocomposites in the absence or presence of UV irradiation for K562 cell.

In the current study, TiO_2_ Ws could be ingested into cancer cells, so photocatalytic attack may occur inside the cancer cells [25]. To explore the possibility of TiO_2_-coated GA with UV as a strategy for comprehensive cancer treatment, the efficiency of GA-TiO_2_ composites under UV irradiation was investigated. It was obvious that there were no significant differences between the purple line and blue line in Figure 5, which represent GA with UV and GA only, respectively, indicating that UV irradiation itself only showed a slightly enhanced effect on K562 cells. MTT assay illustrated that UV irradiation could obviously increase the mortality of K562 cells upon incubation with GA-TiO_2_ nanocomposites than no UV irradiation, as shown in Figure 5 (green line). This finding demonstrates that despite the mortality effects on K562 cells induced by GA, the photocatalytic activity of TiO_2_ Ws could enhance the inhibitation of growth on cells. The IC_50_ value (the half maximal inhibitory concentration of a substance) was determined from the dose–response relationship (Figure 5, inset). The IC_50_ value of free GA was 1.41 mg/mL for the cancer cells; GA with UV and GA-TiO_2_ composites could alter the IC_50_ value to 1.35 and 0.80 mg/mL, respectively. When target cells were treated with GA-TiO_2_ nanocomposites with UV, the IC_50_ value could even be reduced to 0.39 mg/mL. Considering that the serious side effects of GA are related to the high dose of it, the lower IC_50_ of GA-TiO_2_ nanocomposites and GA-TiO_2_ nanocomposites with UV irradiation improved the efficacy on cancer therapy without high concentration of GA and minimized its toxic side effects.

### GA-TiO_2_ nanocomposites significantly induced K562 cell apoptosis

**Figure 6:**
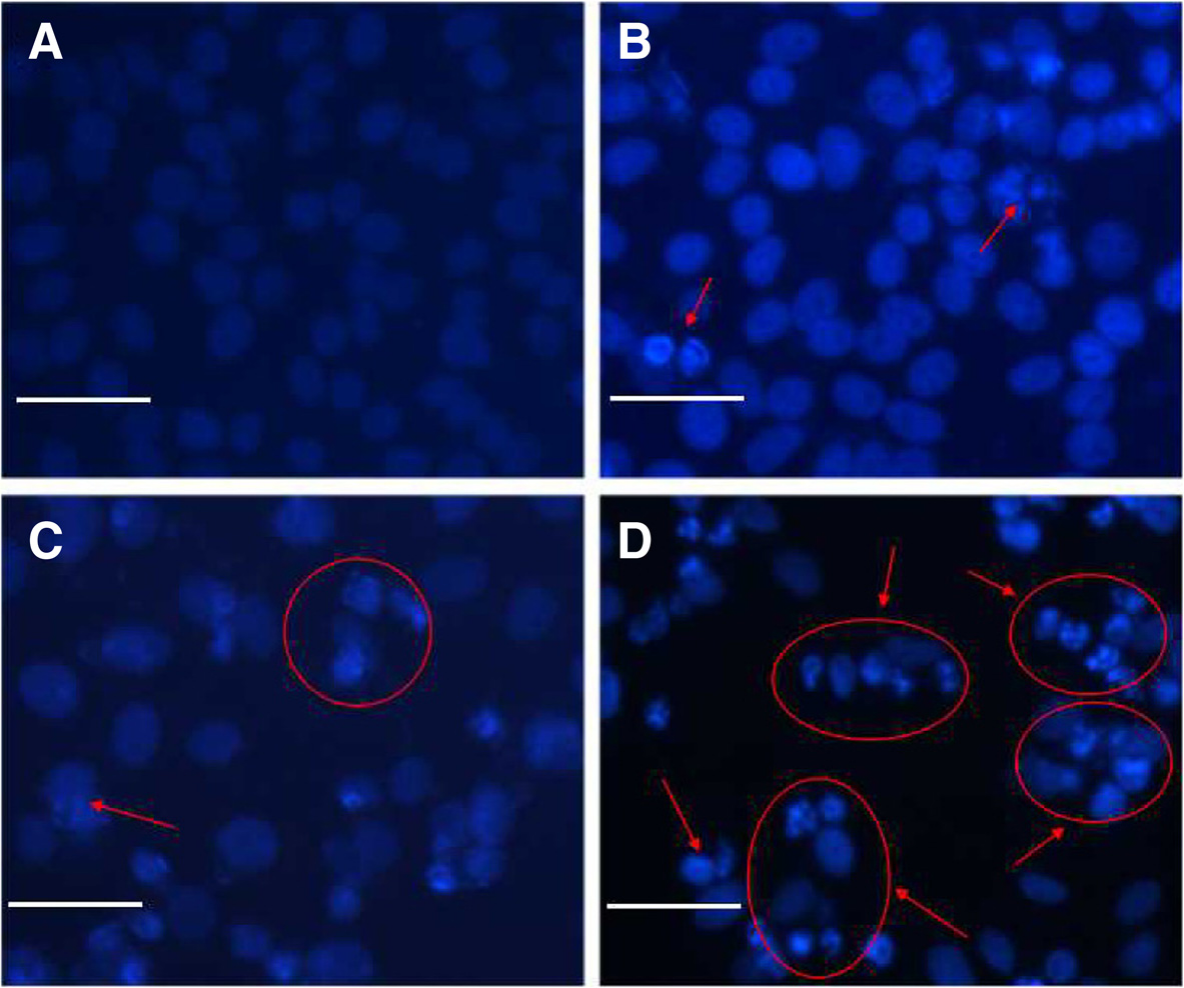
**The fluorescence microscopy images of K562 cells.** Notes: **(A)** treated with 1 μg/mL GA; **(B)** 10 μg/mL TiO_2_ Ws; **(C)** and the nanocomposites with 1 μg/mL GA conjugated with 10 μg/mL TiO_2_ Ws; **(D)** with UV irradiation after DAPI dyeing. Scale bar: 20 μm.

Further study was carried out to observe the morphological changes of K562 cells. Optical microscopy demonstrated the changes in the morphology of K562 cells in different experimental conditions. The evaluation of normal or apoptotic cells depends on morphological characterization. Normal nuclei (smooth nucleus) and apoptotic nuclei (condensed or fragmented chromatin) were easily distinguished. Under fluorescence microscopy, K562 cells in the control group (treated with 1 μg/mL GA) were dyed equally with blue fluorescence, indicating the equivalent distribution of chromatin in the nucleolus, as shown in Figure 6A. By contrast, when treated with 10 μg/mL TiO_2_ Ws, GA induced a few K562 cells to display chromatin condensation and nucleolus pyknosis (Figure 6B). Figure 6C shows that some cancer cells were necrotic with the presence of GA-TiO_2_ nanocomposites, which was conjugated by 1 μg/mL GA and 10 μg/mL TiO_2_ Ws. As shown in Figure 6D, after incubation with GA-TiO_2_ nanocomposites under UV irradiation for 24 h, the cells emitting bright fluorescence increased and displayed the typical appearances of apoptosis, including chromatin condensation, nucleolus pyknosis, nuclear fragmentation, and necrosis. Thus, with the assistance of UV irradiation, cancer cells were killed by the GA-TiO_2_ nanocomposites in the method of apoptosis instead of necrosis.

**Figure 7:**
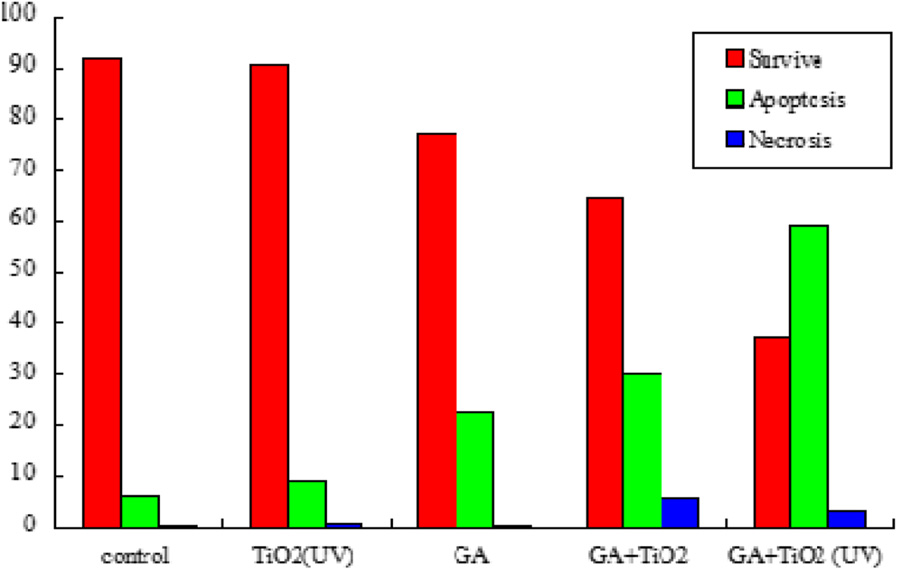
**Effect of GA, TiO**_**2**_ **Ws, and nanocomposites between GA- and TiO**_**2**_ **-induced apoptosis in K562 cells for 24 h.** Notes: the apoptosis analysis of K562 cells in which K562 cells, K562 incubated with 10 μg/mL TiO_2_ Ws, K562 incubated with 1 μg/mL GA, K562 incubated with the nanocomposites with 1 μg/mL GA conjugated with 10 μg/mL TiO_2_ Ws, and K562 incubated with GA-TiO_2_ nanocomposites under UV irradiation.

Then, the flow cytometry was applied to quantitatively investigate the apoptosis of K562 cells. After cells were cultured for 24 h, the total apoptosis rate was 6.6% in the blank experiments which can be seen in Figure 7 and Additional file 1: Figure S1a. When cultured with 1 μg/mL of GA, the early apoptosis and late apoptosis rates respectively increased to 17.6% and 5.3% (Figure 7), and these rates were not significantly different from those in the blank experiments. Combined with TiO_2_ Ws and UV irradiation, an apoptosis rate of approximately 9.0% for K562 was observed. As shown in Figure 7 and Additional file 1: Figure S1, the early apoptosis rate increased to 11.4% and the late apoptosis rate increased to 18.5%, with a total apoptosis rate increased to 29.9% after the participation of GA-TiO_2_ nanocomposites in K562 cells. The apoptosis rate of cells further increased to 59.2% with the application of UV irradiation in the above system, which shows a tremendous change compared with that in the control group (*P* < 0.05).

Depending on all the above observations, a conclusion that TiO_2_ Ws could significantly enhance the cytotoxicity of GA for K562 cells as a drug carrier can be drawn. In comparing with the negative control of GA, cell mortality increased in the presence of GA-TiO_2_ nanocomposites. Apart from acting as excellent drug carriers, TiO_2_ Ws could also serve as good photosensitizers, which perform a great potential for PDT to kill cancer cells effectively. After UV irradiation is applied on the drug nanocomposites, cell viability considerably decreased.

### Effect of GA and GA-TiO_2_ nanocomposites on the cell cycle by flow cytometry

**Figure 8:**
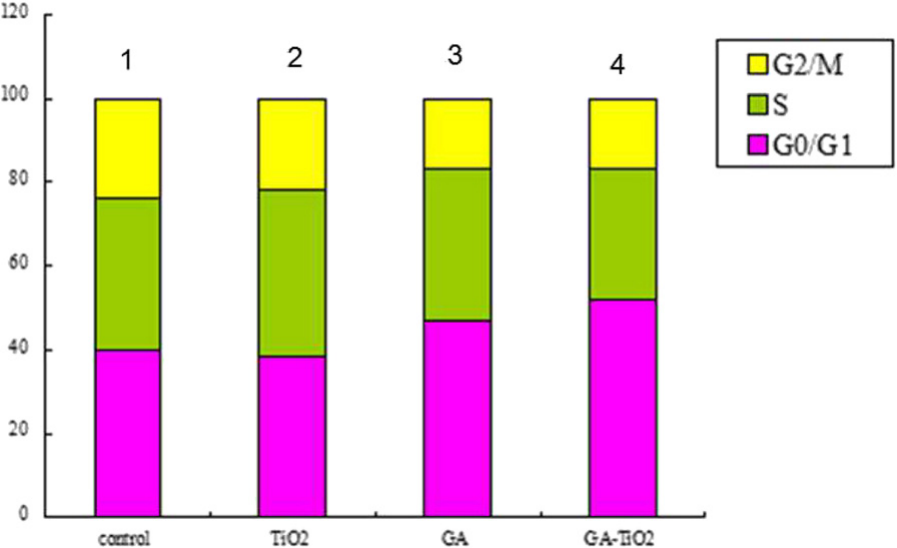
**Effect of GA, TiO**_**2**_ **Ws, and nanocomposites for K562 cells’ cycle under UV irradiation.** Notes: effect of GA, TiO_2_ Ws, and GA-TiO_2_ nanocomposites for K562 cells’ cycle; (1) K562 cells; (2) K562 incubated with 10 μg/mL TiO_2_ Ws; (3) K562 incubated with 1 μg/mL GA; (4) K562 incubated with GA-TiO_2_ nanocomposites (UV) for 24 h.

To investigate the relative mechanism of different systems, cell cycle study was conducted. According to a previous report, GA can induce G0/G1 arrest and K562 apoptosis [26]. As shown in Additional file 1: Figure S2A, the ratio of the G0/G1 phase was approximately 39.90% in the blank experiments of K562 cells, whereas the ratio of the S phase was approximately 36.52%. These results indicate that TiO_2_ Ws had a small effect on the K562 cell cycle, with a ratio of 38.25% in the G0/G1 phase and 39.84% in the S phase (Additional file 1: Figure S2B). The ratio of the S phase decreased to 36.26%, whereas that of the G0/G1 phase increased to 46.88% (Additional file 1: Figure S2C) after the cells were cultured with GA for 24 h. When the K562 cells were cultured with GA-TiO_2_ nanocomposites for 24 h, the G0/G1 phase increased to 52.26% and the S phase decreased to 30.97% (Additional file 1: Figure S2D). The comparison of the effects of GA, TiO_2_ Ws, and GA-TiO_2_ nanocomposites on the K562 cell cycle was shown in Figure 8. An obvious arrest by approximately 5.38% for the G0/G1 phase was observed compared with the GA-treated system. Therefore, the GA-TiO_2_ nanocomposites could increase the cytotoxicity of GA, leading to the significant inhibitation of the growth of K562 cells by perturbation of the cycle signaling network (through the G0/G1 phase).

### Activity of caspase-3, CDK2, CDK4, cyclin D1, p21, and p27 during induced apoptosis

To explore the preliminary apoptotic mechanisms, the changes in the expression levels of the apoptosis regulatory proteins (e.g., caspase-3, CDK2, CDK4, cyclin D1, p21, and p27) were examined by RT-PCR and Western blot.

**Figure 9:**
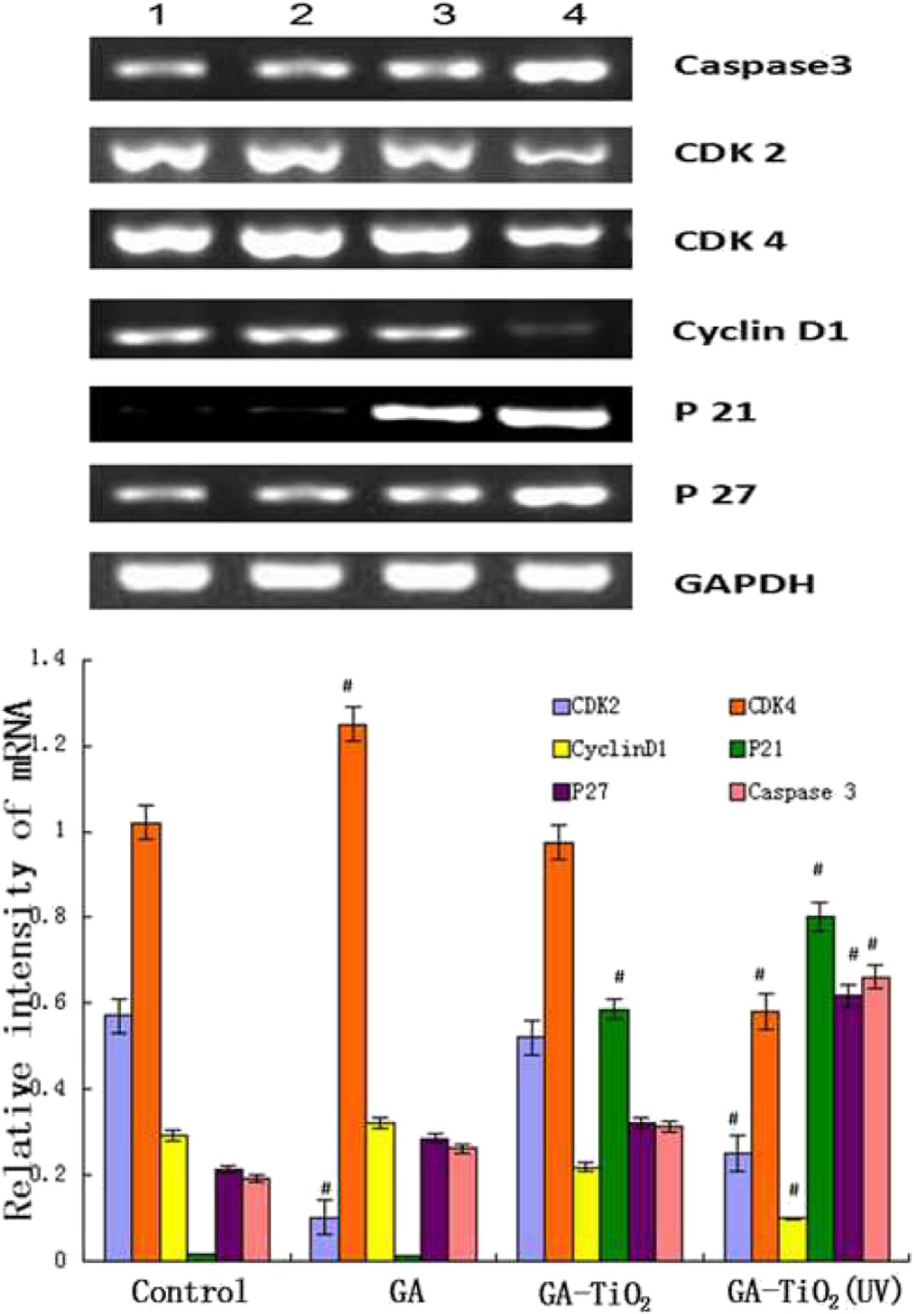
**mRNA expression of apoptosis-associated genes by RQRT-PCR.** K562 cells were treated with different reagents for 24 h. Notes: data were normalized to K562 cell blank group. (1) K562 cells were incubated with same volume of saline; (2) K562 cells were treated with GA; (3) K562 cells were incubated with GA-TiO2 nanocomposites; (4) K562 cells were incubated with GA-TiO_2_ nanocomposites with UV irradiation; data were figured as mean ± SD. ^#^ *P* < 0.05 when compared with the control group.

**Figure 10:**
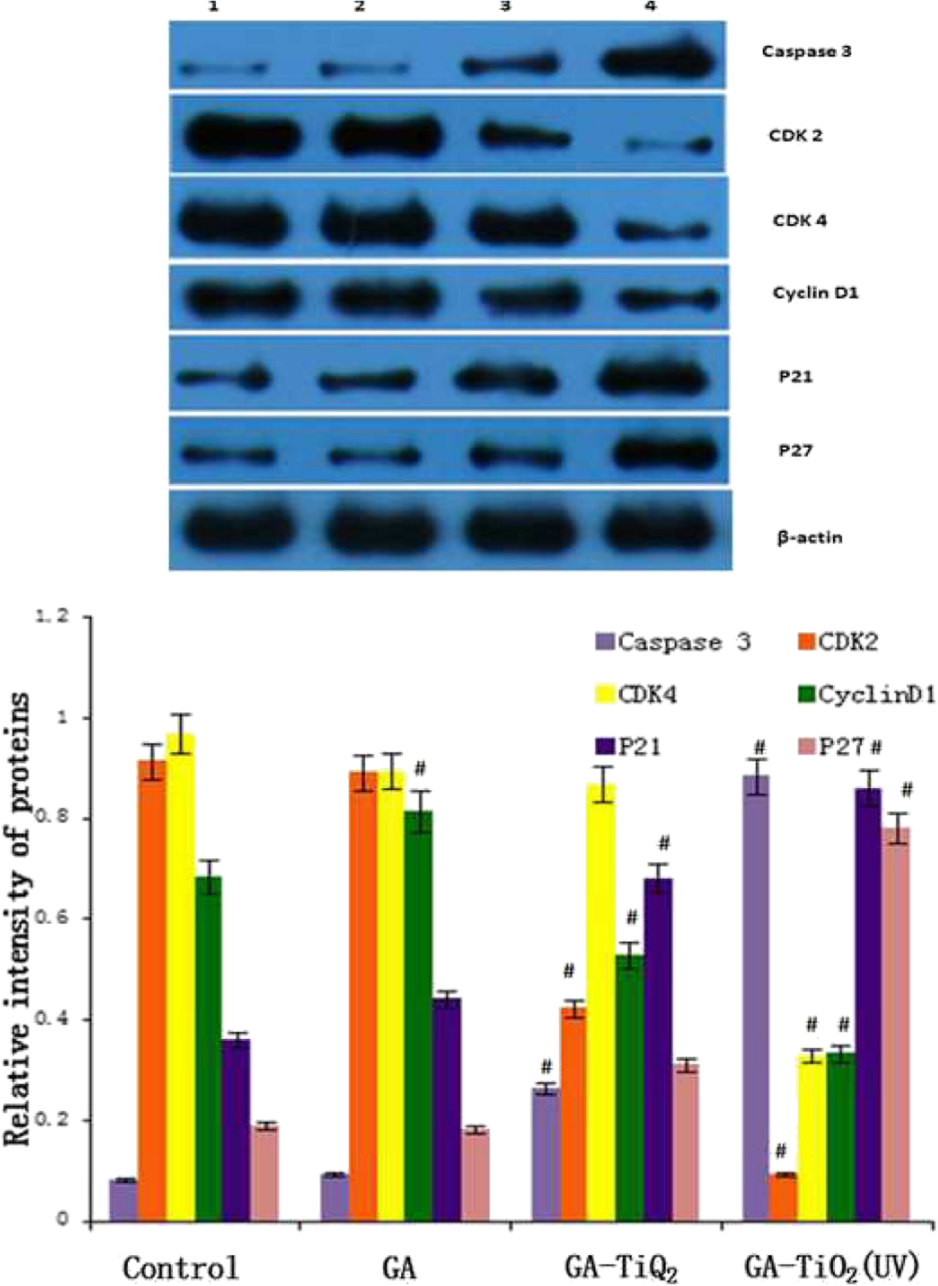
**Protein expression of apoptosis-associated genes by Western blot.** K562 cells were treated with different reagents for 24 h. Notes: data were normalized to β-actin expression. (1) K562 cells were incubated with same volume of saline; (2) K562 cells were treated with GA; (3) K562 cells were incubated with GA-TiO2 nanocomposites; (4) K562 cells were incubated with GA-TiO_2_ nanocomposites with UV irradiation; data were figured as mean ± SD. ^#^ *P* < 0.05 when compared with the control group.

In cell proliferation, cyclin D1 displays the crucial function, which ensures the cell access to S period from G1 period. With the combination of cyclin D1 and CDK4, leading to the activation of CDK4, the activated CDK4 promotes the phosphorylation of the downstream pRb, in which way advances the cell cycle crossing over checkpoints and leads to abnormal proliferation. CDK2 combines with cyclin E as a complex and phosphorylates the downstream Rb, which induces the progression of cell cycle. P21 and P27 play an important role in inducing apoptosis of cells by competitively inhibiting the cyclin or cyclin-CDK, resulting in the loss of biological function of cyclin. PARP is the most important substrate of caspase-3, which is associated with DNA repair and monitoring of genetic integrity. PARP cannot develop its normal function when it is cut into two fragments by caspase-3, finally eliciting the apoptosis of cells. So we can see from Figure 9 that compared with the control group, transcription of CDK2, CDK4, and cyclin D1 messenger RNA (mRNA) was downregulated, and the expression levels of caspase-3, p21, and p27 were upregulated to some extent in GA, GA-TiO_2_ nanocomposites, and GA-TiO_2_ nanocomposites with UV irradiation. The same conclusion can be drawn from Figure 10. These results indicate that photodynamic TiO_2_ Ws could load GA to induce a marked improvement in antitumor activity in various apoptotic pathways.

**Figure 11:**
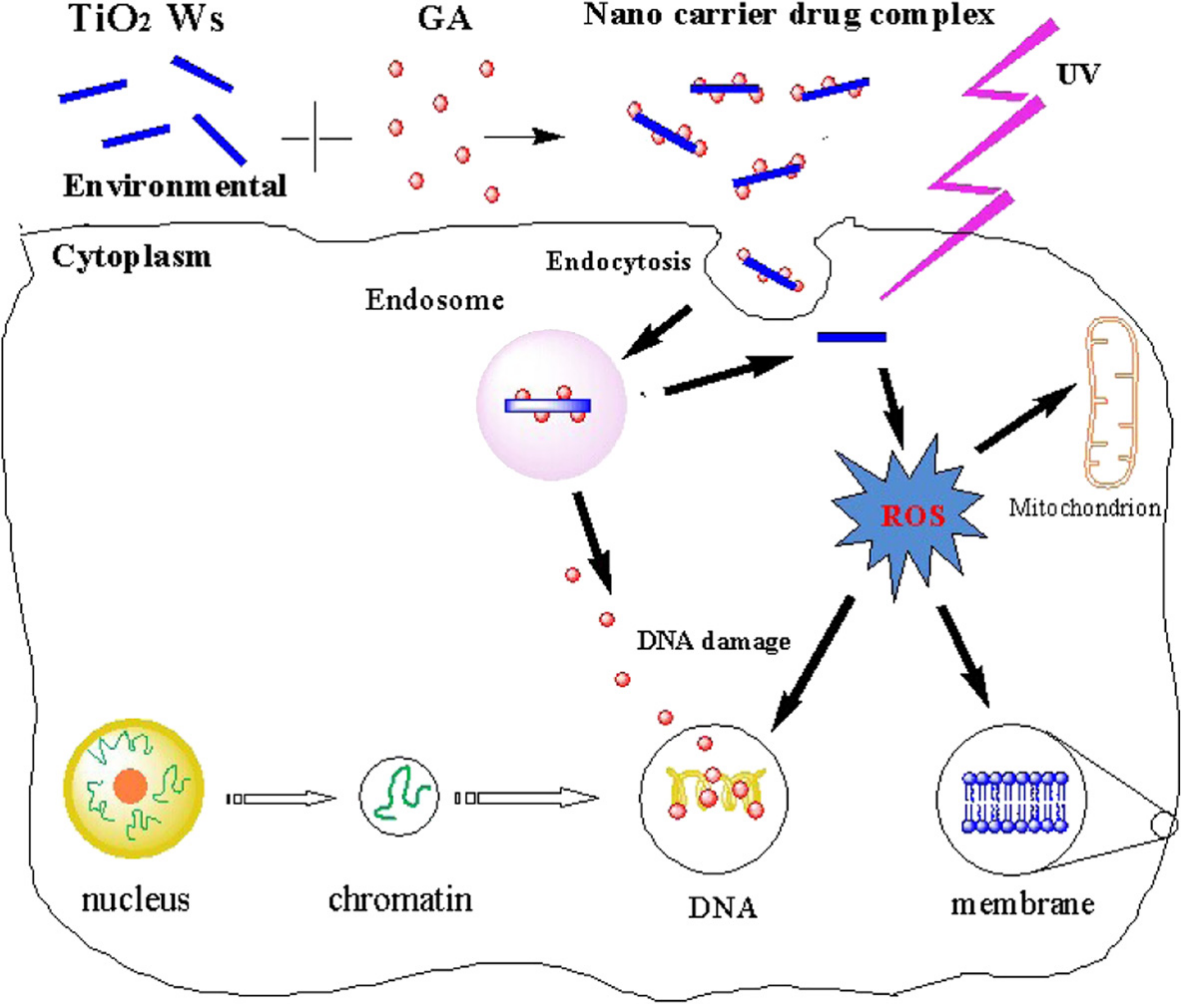
**Improvement in antitumor activity by photodynamic TiO**_**2**_ **Ws loading GA.** Schematic illustration of the possible mechanism of distinguished improvement in antitumor activity by photodynamic TiO2 Ws-loading GA.

In consequence, on the basis of the above results, Figure 11 schematically illustrates the possible processes of photodynamic TiO_2_ Ws coating GA to induce the improvement in antitumor activity remarkably*.* Firstly, TiO_2_ Ws can efficiently uptake GA for the unique high-area properties of them. Secondly, nanocarrier drug composites have the dual functions: they act as drug carriers to deliver GA into cancer cells and function as TiO_2_ Ws under UV irradiation for PDT at the same time. With UV irradiation, TiO_2_ Ws can generate ROS, which can induce the apoptosis of cells. Therefore, TiO_2_ Ws can increase the intracellular concentration of GA dramatically and enhance the suppression of cancer cell proliferation. Meanwhile, the excellent photocatalytic activity of TiO_2_ Ws could enhance the proliferation suppression ability of GA on K562 cells, showing their great potential in PDT. Thus, TiO_2_ Ws exhibit an enormous potential in the application of cancer therapy.

## Conclusion

In this study, we explored the potential application of coupling TiO_2_ Ws with anticancer drug GA in PDT for the first time. The results demonstrate that TiO_2_ Ws have the feature of uniform diameter distribution and high degree of crystallinity, and they could be a kind of safe and efficacious photosensitizer. Moreover, TiO_2_ Ws could enhance the efficacy and lower down the side effects of GA. Therefore, GA-TiO_2_ composites in PDT can be a great potential solution for comprehensive cancer treatment in clinical.

## Additional file

Additional file 1: Figure S1. Effect of GA, TiO_2_ Ws, and nanocomposites between GA- and TiO_2_-induced apoptosis in K562 cells for 24 h. a) Control; b) incubated with 10 μg/ml TiO_2_; (c) incubated with 1 μg/ml GA; (d) incubated with 1 μg/ml GA and 10 mg/L TiO_2_; (e) incubated with GA-TiO_2_ nanocomposites for UV irradiation. **Figure S2.** Effect of GA, TiO_2_ Ws, and nanocomposites for K562 cells’ cycle under UV irradiation. (A) Control; (B) incubated with GA; (C) incubated with TiO2 Ws; (D) incubated with nanocomposites for 24 h.

## Abbreviations

GA: gambogic acid
TiO_2_ Ws: titanium dioxide whiskers
GA-TiO_2_: nanocomposites based on gambogic acid (GA) and titanium dioxide (TiO_2_) whiskers (TiO_2_ Ws)
IC50: half maximal inhibitory concentration
