# Supplementary material for: A new strategy for TiO2 whiskers mediated multi-mode cancer treatment
Source: Nanoscale Res Lett. 2015 Feb 28;10:94. doi: 10.1186/s11671-015-0796-4 (PMC4385221; doi:10.1186/s11671-015-0796-4)
Supplement: Additional file 1: Figure S1. — Effect of GA, TiO2 Ws, and nanocomposites between GA- and TiO2-induced apoptosis in K562 cells for 24 h. a) Control; b) incubated with 10 μg/ml TiO2; (c) incubated with 1 μg/ml GA; (d) incubated with 1 μg/ml GA and 10 mg/L TiO2; (e) incubated with GA-TiO2 nanocomposites for UV irradiation. Figure S2. Effect of GA, TiO2 Ws, and nanocomposites for K562 cells’ cycle under UV irradiation. (A) Control; (B) incubated with GA; (C) incubated with TiO2 Ws; (D) incubated with nanocomposites for 24 h. [file 11671_2015_796_MOESM1_ESM.doc]

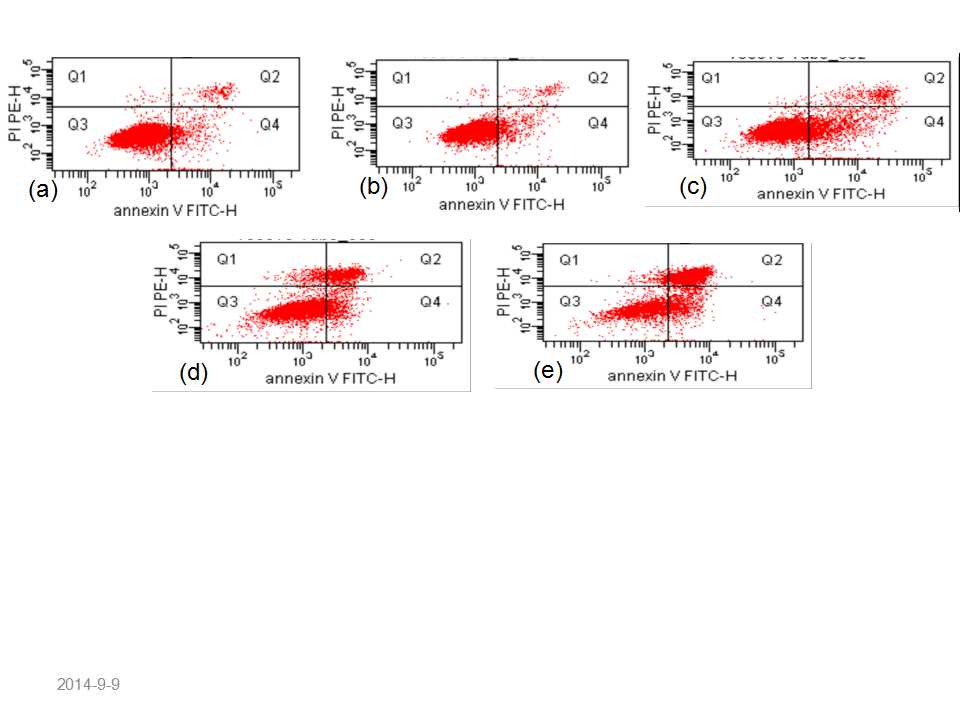


**Fig****.** **S1.** Effect of GA, TiO_2_ Ws, and nanocomposites between GA and TiO_2_ induced apoptosis in K562 cells for 24 h. a) Control; b) Incubated with 10 µg/ml TiO_2_; (c) Incubated with1 µg/ml GA; (d) Incubated with 1 µg/ml GA and 10 mg/L TiO_2_; (e) Incubated with GA - TiO_2_ nanocomposites for UV irradiation.

A

B

C

D

**Fig. S2.** Effect of GA, TiO_2_ Ws, and nanocomposites for K562 cells’ cycle under UV irradiation. (A) Control; (B) Incubated withGA; (C) Incubated with TiO2 Ws; (D) Incubated with nanocomposites for 24 h.
